# Commentary: Beyond C4: Analysis of the complement gene pathway shows enrichment for IQ in patients with psychotic disorders and healthy controls

**DOI:** 10.3389/fimmu.2019.02853

**Published:** 2019-12-04

**Authors:** Liam G. Coulthard, Trent M. Woodruff

**Affiliations:** ^1^Kenneth G Jamieson Department of Neurosurgery, Royal Brisbane and Women's Hospital, Brisbane, QLD, Australia; ^2^Faculty of Medicine, The University of Queensland, St Lucia, QLD, Australia

**Keywords:** complement - immunological terms, neurodevelopment, neuroimmunology, C5aR, synaptic pruning, SERPING1, schizophrenia

Our interest was piqued by the recent study presented in *Genes, Brain and Behavior* by Holland et al. (1). They outline an important investigation highlighting the link between complement gene expression and IQ, in both schizophrenic patients and healthy controls. The study demonstrated a broad association between variations in complement gene expression and perturbed neurodevelopment. We were particularly interested to see that the final sentence of the paper stated that “*When complement gene-sets are taken as a whole, their relevance is to neurodevelopment, not illness.”* Their conclusion rightly touches on the emerging understanding of non-immune roles for complement in diverse biological processes (2).

The complement group of proteins is ancient in evolutionary terms. Recognized complement components are found throughout the animal kingdom including in rudimentary diploblastic species (3). In the human adult, complement functions as an essential pillar of the innate immune system. It is activated by various noxious stimuli and results in the production of the anaphylatoxins and membrane attack complex (4). Expectedly, the human collection of complement factors 1–9, associated factors, receptors and regulators is not replicated within jellyfish and their like. These more simple creatures express opsonins, such as C3, which have historically been thought to function in a basic immune system (3). Whether the more recently discovered, novel roles of complement apply in these ancient species is unknown (5). In the developing mammal we know that the proteins of the complement system are both temporally and spatially separate in their expression (6, 7). This, we think, underscores the developmental importance of the other roles of the complement proteins in the absence of the established pathways of activation. There are now several decades of good evidence of separate and distinct roles for complement proteins in both neurodevelopment and general development/regeneration.

In the work by Holland and colleagues, they expand on previous research demonstrating a link between complement and pathogenesis of schizophrenia (1). A recent seminal paper in the fields showed SNPs contributing to altered C4 protein expression resulting in an increased risk of schizophrenia. Interestingly, the risk conferred by each SNP was proportional to the magnitude of *C4A* allele overexpression, but not *C4B*. This perhaps indicating that there may be subtle differences in the functions of the C4 alleles that become most apparent in neurodevelopment (8). In addition to C4, other complement factors, such as *C5* and *SERPING1* have also been shown to be associated with a thin frontal cortex, thought to be pathogenic for schizophrenia (9). Holland and colleagues performed a gene-set analysis, looking specifically for the complement-related genes that are associated with IQ and schizophrenia. Intriguingly, despite previous evidence of individual complement gene association with schizophrenia, there was no whole gene-set association with the disease as determined by MAGMA analysis (*n* = 105,318). There was however a gene-set association with IQ (*n* = 269,867, 14 separate cohorts) and 12 individual genes showed a significant individual association with IQ through polygenic score regression analysis in a smaller independent dataset (*n* = 1,000). Regulators of complement action were heavily represented in this group, with *SERPING1*, a regulator of complement and other plasma enzymes (10), showing the highest association (*p* = 3.97E-09).

To explain their findings, the authors touch upon a key developmental function of the complement system: synaptic pruning. In this role the classical pathway of complement activation acts to selectively tag underactive synapses for removal by microglia (11). Dysregulation can lead to unrefined neural networks in development, or loss of important synapses in degenerative disease (12). The authors are certainly correct that this may be a key mechanism leading to variations in IQ in their samples, however here we highlight other mechanisms that may also contribute.

Neuronal migration during neocortical development is a process dependent on proteins of the lectin pathway of complement activation. Knockdown of *MASP1, C3*, and *SERPING1* results in ectopic migrating neuroblasts, a phenotype rescued by forced expression of a downstream split product of C3 (13, 14). The effect of complete knockdown is a dysregulated cortical layering, which would presumably lead to impaired behavioral phenotypes in the resultant adult mice. It is plausible that subtle perturbation of migration, through variations in expression of these genes, may lead to subtle impairments in cognitive development. Perhaps this could account for the *SERPING1* association found in the work of Holland et al. (1).

Our own recent findings have shown key roles for the anaphylatoxin receptors, C5aR1, and C3aR, in directing neural progenitor cell fate. Both receptors are expressed within neural tissue throughout development, from neurulation onwards (15, 16). Here the anaphylatoxin receptors are polarized to the ventricular surface of neural progenitor cells (6, 15, 17). Despite their similar location, the functions of C3aR and C5aR1 seem to be diametrically opposed; C5aR1 promotes polarity and therefore proliferation of neural progenitors, whereas C3aR promotes differentiation (15, 17).

C3aR and C5aR1 knockout mice exhibit grossly normal physical development, though show cognitive deficits when compared to wild-type controls, both demonstrating impairments in memory (15, 17). It is unclear whether this is a result of perturbations to complement-associated roles dictating progenitor fate, migration or synaptic pruning during development (or even due to complement actions in the adult brain). Similarly, the results of Holland and colleagues, where there is association of multiple complement regulator genes with IQ, cannot be attributed solely to one of these developmental roles. Predicting the likely mechanism is fraught with difficulty, not least because the complement system represents a complex web of overlapping pathways and regulators. The common convergence point of all complement pathways remains C3, with the generation of C3a and C5a anaphylatoxins. Therefore, there still exists a possibility that the phenotypes exhibited in the knockout animals, and in the report that forms the subject of this commentary, could be the result of any or all of these novel roles.

To conclude, we commend the work by Holland and colleagues for demonstrating a fascinating link between complement gene polymorphisms and IQ. In the current study they found that *SERPING1* had the highest association with IQ (1) and several other key regulators also showed significant association. The study is an important demonstration of the role of complement proteins in normal neurodevelopment. Most interestingly, the pathophysiological mechanisms between altered gene expression and impaired cognition are not yet clear. There are several potential mechanisms that could hold the possibility of an explanation (18), as outlined briefly in this commentary. We remain excited about the future developments in this field, building on this work, to further define how this versatile group of proteins contributes to the normal neurodevelopment.

## Author Contributions

All authors listed have made a substantial, direct and intellectual contribution to the work, and approved it for publication.

### Conflict of Interest

The authors declare that the research was conducted in the absence of any commercial or financial relationships that could be construed as a potential conflict of interest.
